# Population genetic analysis reveals a low level of genetic diversity of ‘*Candidatus* Phytoplasma aurantifolia’ causing witches’ broom disease in lime

**DOI:** 10.1186/s40064-016-3401-0

**Published:** 2016-10-03

**Authors:** Shaikha Y. Al-Abadi, Abdullah M. Al-Sadi, Matthew Dickinson, Mohammed S. Al-Hammadi, Rashid Al-Shariqi, Rashid A. Al-Yahyai, Elham A. Kazerooni, Assunta Bertaccini

**Affiliations:** 1 Royal Court Affairs, Seeb, Oman; 2 Department of Crop Sciences, College of Agricultural and Marine Sciences, Sultan Qaboos University, PO Box 8, 123 Al Khoudh, Oman; 3 School of Biosciences, University of Nottingham, Sutton Bonington Campus, Loughborough, UK; 4 Abu Dhabi Food Control Authority, PO Box 52150, Abu Dhabi, UAE; 5 Islamic Azad University, Shiraz, Iran; 6 Department of Agricultural Sciences, Plant Pathology, Alma Mater Studiorum-University of Bologna, Bologna, Italy

**Keywords:** WBDL, Phytoplasma, Acid lime, Population structure

## Abstract

Witches’ broom disease of lime (WBDL) is a serious phytoplasma disease of acid lime in Oman, the UAE and Iran. Despite efforts to study it, no systemic study attempted to characterize the relationship among the associated phytoplasma, ‘*Candidatus* Phytoplasma aurantifolia’, from the three countries. This study utilized sequences of the 16S rRNA, *imp* and *secA* genes to characterize 57 strains collected from Oman (38), the UAE (9) and Iran (10). Phylogenetic analysis based on the 16S rRNA gene showed that the 57 strains shared 98.5–100 % nucleotide similarity to each other and to strains of ‘*Ca*. P. aurantifolia’ available in GenBank. The level of genetic diversity was low based on the 16S rRNA (0-0.011), *imp* (0–0.002) and *secA* genes (0–0.015). The presence of low level of diversity among phytoplasma strains from Oman, the UAE and Iran can be explained by the movement of infected lime seedlings from one country to another through trading and exchange of infected plants. The study discusses implication of the findings on WBDL spread and management.

## Background

Citrus fruits are among the most important fruits in the world due to their high nutritional value and possibility to be consumed fresh as well as processed. Orange trees are the most widely cultivated citrus species, with Brazil being the largest orange exporter in the world. Lime and lemon trees are also cultivated extensively around the globe. India, with about 16 % of the world’s overall lemon and lime production, tops the production list, followed by Mexico (~14.5 %), Argentina (~10 %), Brazil (~8 %), and Spain (~7 %) (FAO 2015).

*Citrus aurantifolia* Swingle, known locally as Omani lime, has many other names in other parts of the world, such as Mexican lime, key lime and acid lime. It has been grown in Oman for at least four centuries where it was brought by Arabian sailors (Davies and Albrigo 1994). It was considered as the second most important fruit crop after dates in Oman until the 1970s, and today it is ranked among the top four fruit crops in terms of production. It is also an important crop in the UAE and Iran (FAO 2015).

Witches’ broom disease (WBDL) is the most destructive disease of lime trees in Oman, the UAE and Iran (Chung et al. 2006; Al-Yahyai et al. 2015; Al-Sadi et al. 2012b; Bové et al. 2000) where it killed more than a million lime trees. ‘*Candidatus* Phytoplasma aurantifolia’ is the phytoplasma associated with WBDL (Zreik et al. 1995). Phytoplasmas are phloem-limited bacteria which can be transmitted by phloem feeding insects such as leafhoppers and psyllids. WBDL was first observed in Shinas and Liwa in the northern coast of Al-Batinah governorate in the 1970s, then it spread rapidly to other parts of Oman (Bové et al. 1988). It was reported in the UAE in 1989 and in Iran in the 1990s (Garnier et al. 1991; Bové et al. 2000). WBDL results in the production of a large number of leaves, light green to yellow in color and smaller in size. This is also associated with production of many branches. Leaves on the symptomatic branches usually dry within 1 year of symptom appearance. WBDL symptoms progress on the entire canopy until the whole tree collapses and dies within three to 7 years of first symptom appearance (Al-Yahyai et al. 2015; Al-Sadi et al. 2012b).

The classification system of phytoplasmas is based on RFLP and/or sequencing of 16S rDNA (Schneider et al. 1995), while in the past, phytoplasma strains were differentiated according to their biological properties such as the similarity in symptoms, plant host, and insect vectors. This way of classification was laborious, time-consuming and not reliable. Recently a number of genetic markers have been developed for differentiation among phytoplasma strains, including immunodominant membrane protein (*imp*) and *secA* genes (Siampour et al. 2012; Bekele et al. 2011; Hodgetts et al. 2008).

Despite studies conducted on ‘*Ca*. P. aurantifolia’ in Oman and elsewhere there is a lack of knowledge concerning diversity and genetic relationship among ‘*Ca*. P. aurantifolia’ strains. This study was conducted to investigate the diversity of the strains infecting acid lime in Oman, the UAE and Iran based on 16S rRNA, s*ecA*, and *imp* genes, and to determine the ability of the latter two genes to discriminate ‘*Ca*. P. aurantifolia’ strains from phytoplasmas belonging to other ribosomal groups. Knowledge gained about relationships among the WBDL phytoplasma isolates from the three countries will help in developing plans for management strategies to contain WBDL spreading.

## Results

### Survey and sample collection

**Fig. 1:**
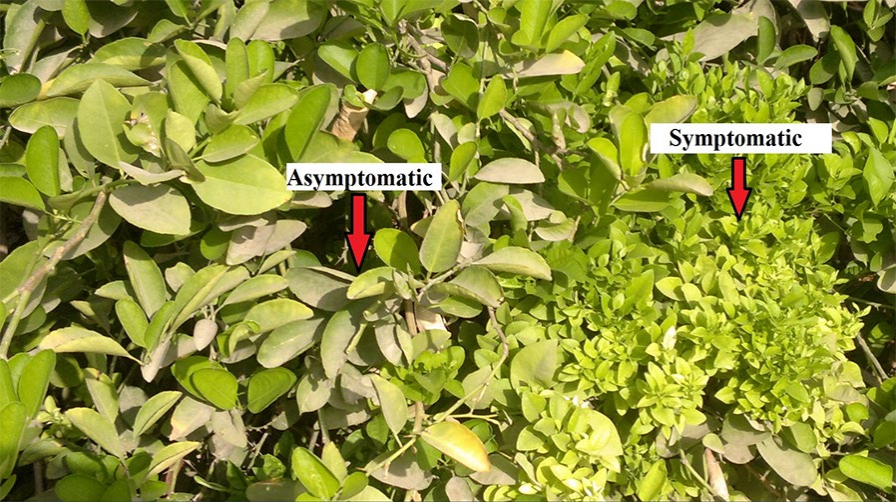
Symptomatic and asymptomatic branches on an acid lime infected by ‘*Ca.* P. aurantifolia’

Surveys in Oman, UAE and Iran showed the presence of typical symptoms of witches’ broom disease in the three countries. Disease symptoms were characterized by appearance of dense growth of shoots, clustering of leaves and branches, and reduction in size and yellowing of leaves (Fig. 1). Dieback symptoms were also observed in one farm in Al-Ain (UAE). Severity of the disease was variable among farms and could not be quantified due to the tendency of many farmers to remove symptomatic branches as soon as they appear. The total samples which were collected during this study were 75 from Oman, 20 from Iran and 20 from the UAE. However, only samples which yielded good quality sequences of the 16S rRNA, *imp* and *secA* genes (38 from Oman, 10 from Iran and 9 from the UAE) were included in further analysis.

### Phytoplasma diversity on 16S rRNA, *secA* and *imp* genes

**Fig. 2:**
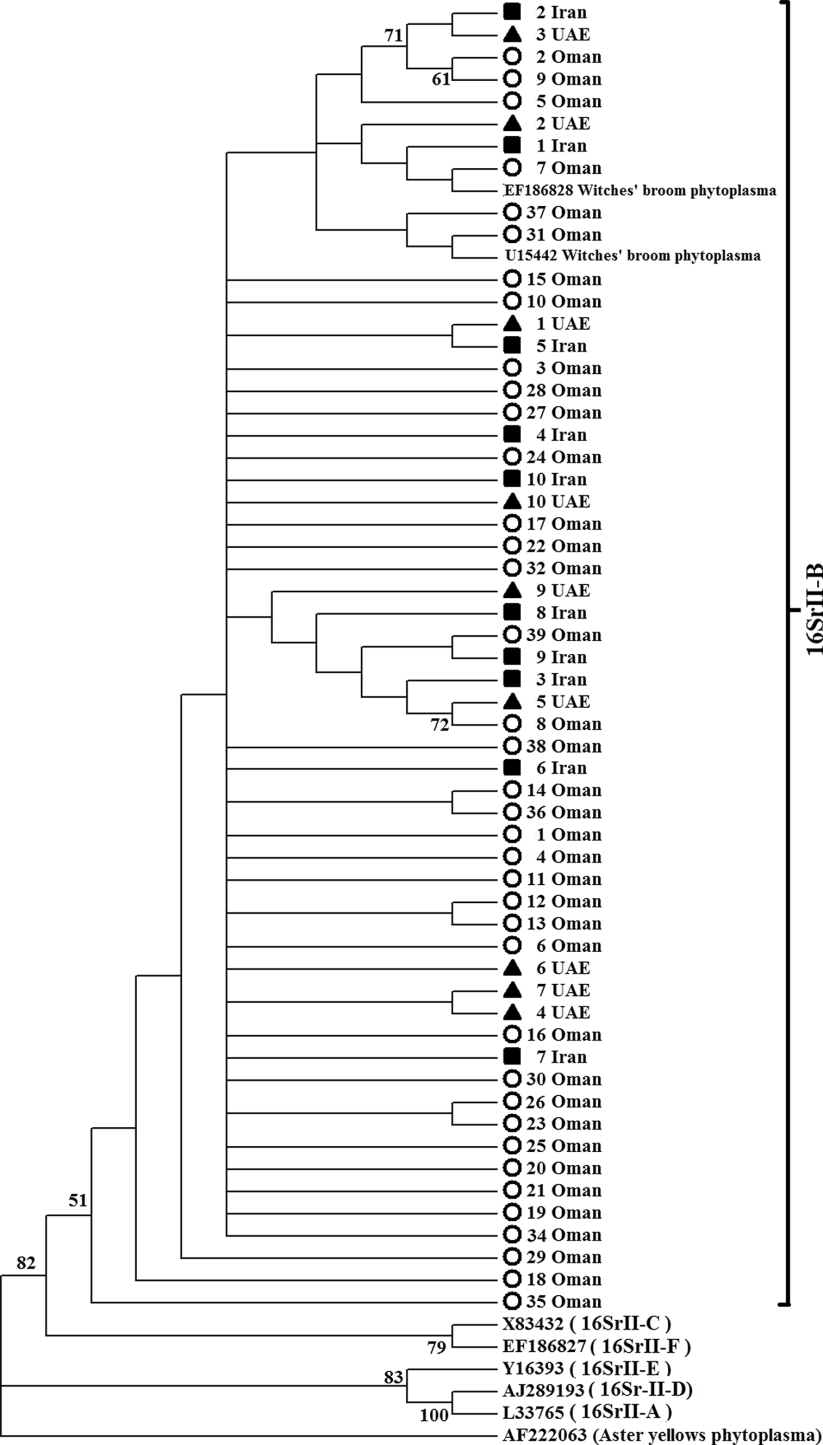
A phylogenetic tree showing analysis of 57 phytoplasma isolates from Oman, UAE and Iran with phytoplasma reference sequences from 16S rRNA Group II. The tree is rooted to ‘*Ca*. Phytoplasma asteris’ (Aster yellows phytoplasma; GenBank no. AF222063). Bootstrap values above 50 % are shown (1000 replications). The *circle*, *triangle* and *square* represent Omani, UAE and Iranian isolates, respectively

PCR analysis of 57 acid lime samples based on the 16-23S rRNA using P1/P7 and R16R2/R16F2n produced fragments of 1784 base pairs (bp) and 1238–1248 bp, respectively (Table 2). Only fragments of 1238 bp representing the 16S rRNA gene of the strains were used in the phylogenetic analysis. Comparison of these sequences showed that strains shared 98.5–100 % (average 99.7 %) nucleotide similarity to each other and 99.4 % similarity to the reference strain of lime witches’ broom phytoplasma from Oman in GenBank (Accession number: EF186828). All strains from Oman clustered with reference strains of ‘*Ca.* P. aurantifolia’ (subgroup 16SII-B) from GenBank (Fig. 2). Analysis of genetic divergence among ‘*Ca.* P. aurantifolia’ strains based on 16S rRNA gene showed that the range of divergence was 0–0.003 for Omani strains, 0–0.002 for UAE strains and 0–0.002 for the Iranian strains (Table 3). The overall level of divergence based on all ‘*Ca*. P. aurantifolia’ from this study and from reference strains was 0.000–0.011 (avg. 0.001).

**Fig. 3:**
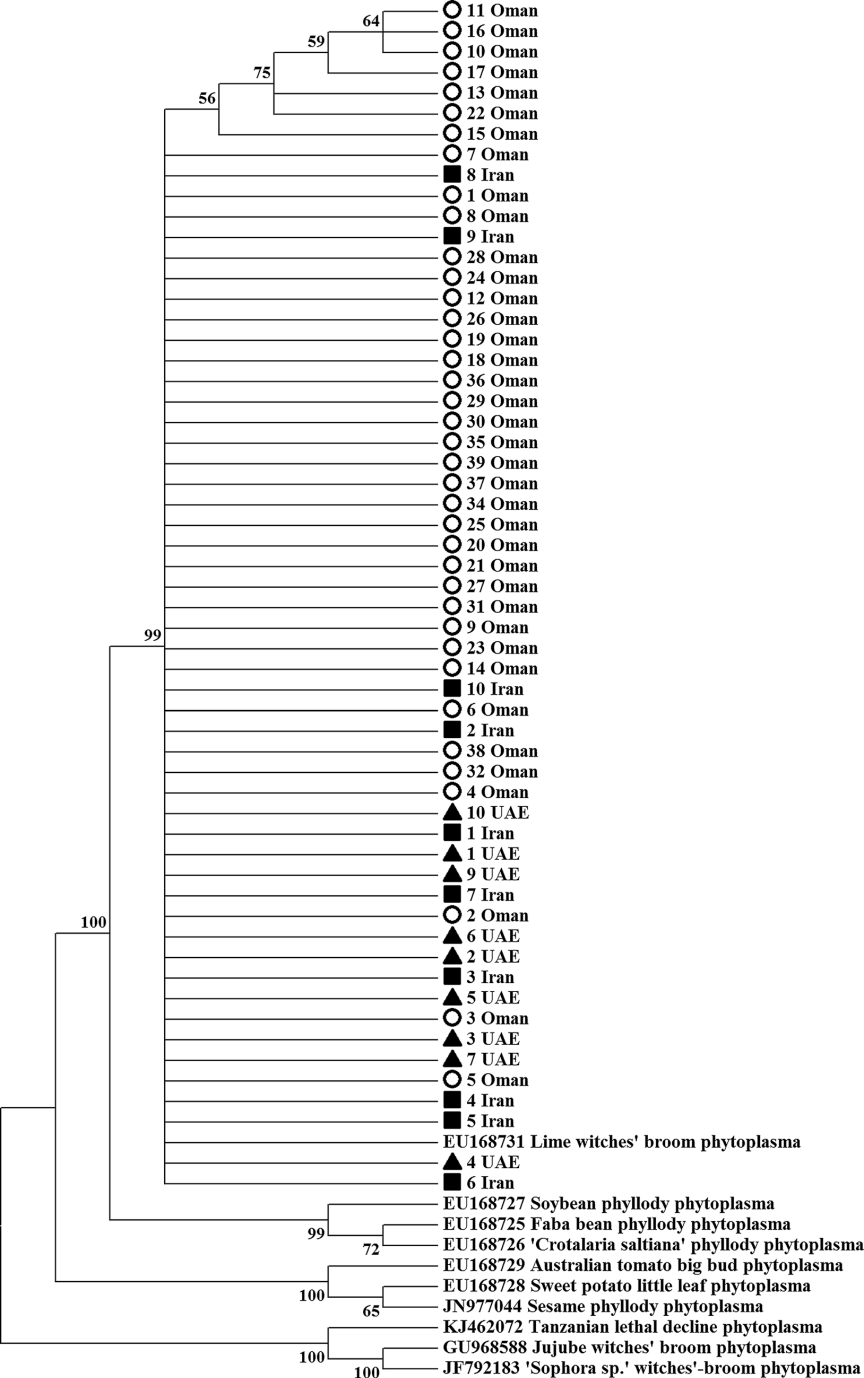
A phylogenetic tree showing analysis of 57 phytoplasma isolates from Oman, the UAE and Iran with 10 *secA* reference sequences of all representative phytoplasmas available in GenBank (phytoplasma sequences with less than 95 % query coverage were excluded). Bootstrap values above 50 % are shown (1000 replications). The *circle*, *triangle* and *square* symbols represent Omani, UAE and Iranian isolates, respectively

The primer pair SecAfor2/SecArev3 resulted in a product of 482 bp in size for the 57 strains analyzed (Table 1). Analysis of s*ecA* sequences showed that all strains share 99.8–100 % sequence similarity to each other and to the *secA* sequence of lime witches’ broom phytoplasma (Accession number: EU168731, Oman). Based on the SecA phylogenetic tree, ‘*Candidatus* Phytoplasma aurantifolia’ isolates were separated from 9 other phytoplasmas with a very high bootstrap support (Fig. 3). Analysis of genetic divergence among the 57 ‘*Ca*. P. aurantifolia’ based on s*ecA* gene sequences showed that the range of divergence is 0-0.010 among Omani strains, 0–0.004 among the UAE strains and 0-0.006 among the Iranian strains (Table 3). The overall sequence divergence in the *secA* genes from all ‘*Ca*. P. aurantifolia’ isolates of this study and the reference strains was 0.000–0.015 (avg. 0.003).

**Table 1:** Primers used for amplifying Phytoplasma genes

Gene	Primer name	5′-3′ Sequence	Product size (bp)	References
16S rRNA	P1	AAGAGTTTGATCCTGGCTCAGGATT	1784	Deng and Hiruki (1991)
	P7	CGTCCTTCATCGGCTCTT		
	R16R2	GAAACGACTGCTAAGACTGG	~1248	Gundersen and Lee (1996)
	R16F2n	TGACGGGTGTGTACAAACCCCG		
*SecA*	SecAfor1	GARATGAAAACTGGRGAAGG	482^a^	Hodgetts et al. (2008)
	SecAfor2	GAYGARGSWAGAACKCCT		
	SecArev3	GTTTTRGCAGTTCCTGTCATNCC		
*imp*	ImpF	ATGAATCACAAAGAAATTTTTTAC	519	Siampour et al. (2012)
	ImpR	TTATGATAATTTTAAATCTG		

^a^Using SecAfor2/SecArev3 primer combinations in a semi-nested PCR (Hodgetts et al. 2008)

**Fig. 4:**
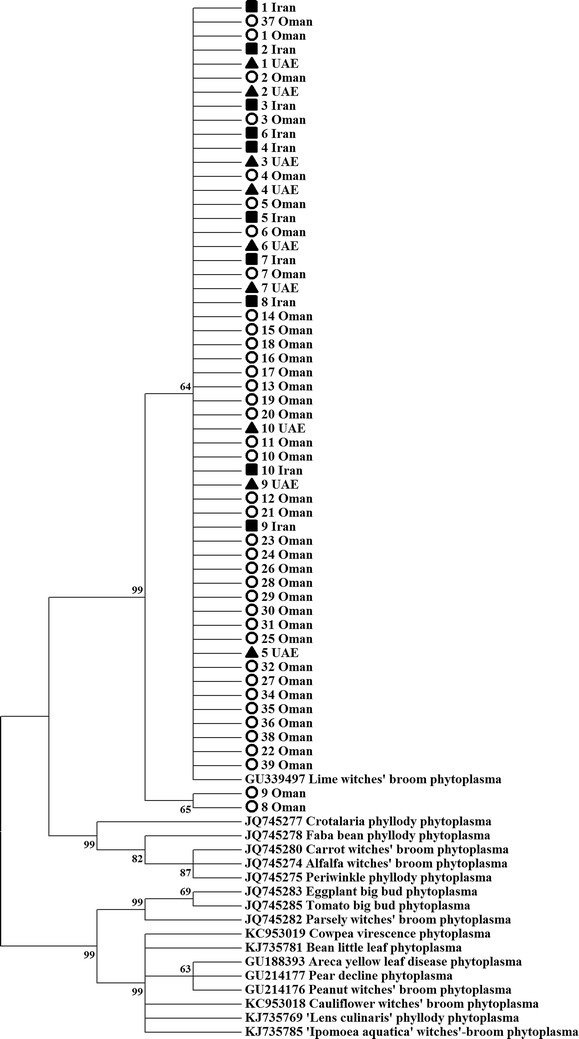
Phylogenetic analysis of 57 phytoplasma isolates from Oman, the UAE and Iran with 17 *imp* reference sequences of all representative phytoplasmas available in GenBank. Bootstrap values above 50 % are shown (1000 replications). The *circle*, *triangle* and *square* symbols represent Omani, UAE and Iranian isolates, respectively

PCR amplification using the primer pair ImpF/ImpR, produced a fragment of 519 bp (Table 1). Analysis based on the *imp* gene sequences showed that 55 phytoplasmas share 100 % sequence similarity to each other and to lime witches’ broom phytoplasma (Accession number: GU339497, Iran), while they were found to share 99.8 % similarity to strains 8 and 9 from Oman (Table 2). Phylogenetic analysis based on the *imp* gene sequences showed that all ‘*Ca*. Phytoplasma aurantifolia’ isolates were separated with a high bootstrap support from 16 other phytoplasmas (Fig. 4). The genetic divergence among the 57 sequences ranged from 0 to 0.002 for the Omani strains, while there was no divergence among the Iranian and UAE strains (Table 3). The overall sequence divergence in the *imp* genes from all ‘*Ca*. P. aurantifolia’ isolates of this study and the reference strains was 0.000–0.015 (avg. 0.003).

**Table 2:** Characteristics of samples collected from Oman, UAE and Iran

No.	Sample code	Country	Governorate/province	Year of collection	GenBank accession numbers		
					16S rRNA	*secA*	*imp*
1	Iran 1	Iran	Hormozgan	2013	LN872963	LN873084	LN873020
2	Iran 2	Iran	Hormozgan	2013	LN872966	LN873087	LN873023
3	Iran 3	Iran	Hormozgan	2013	LN872969	LN873090	LN873026
4	Iran 4	Iran	Hormozgan	2013	LN872972	LN873093	LN873029
5	Iran 5	Iran	Hormozgan	2013	LN872975	LN873096	LN873032
6	Iran 6	Iran	Kerman	2013	LN872978	LN873099	LN873035
7	Iran 7	Iran	Kerman	2013	LN872981	LN873102	LN873038
8	Iran 8	Iran	Kerman	2013	LN872984	LN873105	LN873041
9	Iran 9	Iran	Kerman	2013	LN872986	LN873107	LN873043
10	Iran 10	Iran	Hormozgan	2013	LN872989	LN873110	LN873046
11	UAE 1	UAE	Al-Ain	2013	LN872965	LN873086	LN873022
12	UAE 2	UAE	Al-Ain	2013	LN872968	LN873089	LN873025
13	UAE 3	UAE	Al-Ain	2013	LN872971	LN873092	LN873028
14	UAE 4	UAE	Al-Ain	2013	LN872974	LN873095	LN873031
15	UAE 5	UAE	Al-Ain	2013	LN872977	LN873098	LN873034
16	UAE 6	UAE	Al-Ain	2013	LN872980	LN873101	LN873037
17	UAE 7	UAE	Al-Ain	2013	LN872983	LN873104	LN873040
18	UAE 9	UAE	Al-Ain	2013	LN872988	LN873109	LN873045
19	UAE 10	UAE	Al-Ain	2013	LN872991	LN873112	LN873048
20	Oman 1	Oman	Musandam	2013	LN872964	LN873085	LN873021
21	Oman 2	Oman	Musandam	2013	LN872967	LN873088	LN873024
22	Oman 3	Oman	Musandam	2013	LN872970	LN873091	LN873027
23	Oman 4	Oman	Musandam	2013	LN872973	LN873094	LN873030
24	Oman 5	Oman	Musandam	2013	LN872976	LN873097	LN873033
25	Oman 6	Oman	Musandam	2013	LN872979	LN873100	LN873036
26	Oman 7	Oman	Musandam	2013	LN872982	LN873103	LN873039
27	Oman 8	Oman	Musandam	2013	LN872985	LN873106	LN873042
28	Oman 9	Oman	Musandam	2013	LN872987	LN873108	LN873044
29	Oman 10	Oman	Batinah	2014	LN872990	LN873111	LN873047
30	Oman 11	Oman	Batinah	2014	LN872992	LN873113	LN873049
31	Oman 12	Oman	Batinah	2014	LN872993	LN873114	LN873050
32	Oman 13	Oman	Batinah	2014	LN872994	LN873115	LN873051
33	Oman 14	Oman	Batinah	2014	LN872995	LN873116	LN873052
34	Oman 15	Oman	Batinah	2014	LN872996	LN873117	LN873053
35	Oman 16	Oman	Batinah	2014	LN872997	LN873118	LN873054
36	Oman 17	Oman	Batinah	2014	LN872998	LN873119	LN873055
37	Oman 18	Oman	Batinah	2014	LN872999	LN873120	LN873056
38	Oman 19	Oman	Batinah	2014	LN873000	LN873121	LN873057
39	Oman 20	Oman	Batinah	2014	LN873001	LN873122	LN873058
40	Oman 21	Oman	Batinah	2014	LN873002	LN873123	LN873059
41	Oman 22	Oman	Batinah	2014	LN873003	LN873124	LN873060
42	Oman 23	Oman	Batinah	2014	LN873004	LN873125	LN873061
43	Oman 24	Oman	Batinah	2014	LN873005	LN873126	LN873062
44	Oman 25	Oman	Batinah	2014	LN873006	LN873127	LN873063
45	Oman 26	Oman	Batinah	2014	LN873007	LN873128	LN873064
46	Oman 27	Oman	Dakhlia	2014	LN873008	LN873129	LN873065
47	Oman 28	Oman	Dakhlia	2014	LN873009	LN873130	LN873066
48	Oman 29	Oman	Dakhlia	2014	LN873010	LN873131	LN873067
49	Oman 30	Oman	Dakhlia	2014	LN873011	LN873132	LN873068
50	Oman 31	Oman	Dakhlia	2014	LN873012	LN873133	LN873069
51	Oman 32	Oman	Dakhlia	2014	LN873013	LN873077	LN873070
52	Oman 34	Oman	Dhahira	2014	LN873014	LN873078	LN873071
53	Oman 35	Oman	Dhahira	2014	LN873015	LN873079	LN873072
54	Oman 36	Oman	Dhahira	2014	LN873016	LN873080	LN873073
55	Oman 37	Oman	Dhahira	2014	LN873017	LN873081	LN873074
56	Oman 38	Oman	Dhahira	2014	LN873018	LN873082	LN873075
57	Oman 39	Oman	Dhahira	2014	LN873019	LN873083	LN873076

**Table 3:** Estimation of evolutionary divergence among phytoplasma sequences

	Iran			UAE			Oman		
	16S rDNA	*imp*	*sec*A	16S rDNA	*imp*	*sec*A	16S rDNA	*imp*	*sec*A
Mini	0.000	0.000	0.000	0.000	0.00	0.000	0.000	0.000	0.000
Max	0.002	0.000	0.006	0.002	0.00	0.004	0.003	0.002	0.010
Avg.	0.000	0.000	0.002	0.001	0.00	0.001	0.001	0.000	0.003

The table provides estimates of evolutionary divergence over sequence pairs within phytoplasma groups from the different countries. The analysis involved 57 nucleotide sequences. Analyses were conducted using the Kimura 2-parameter model (Mega 6)

### Concatenated sequence analysis of phytoplasma genes

**Fig. 5:**
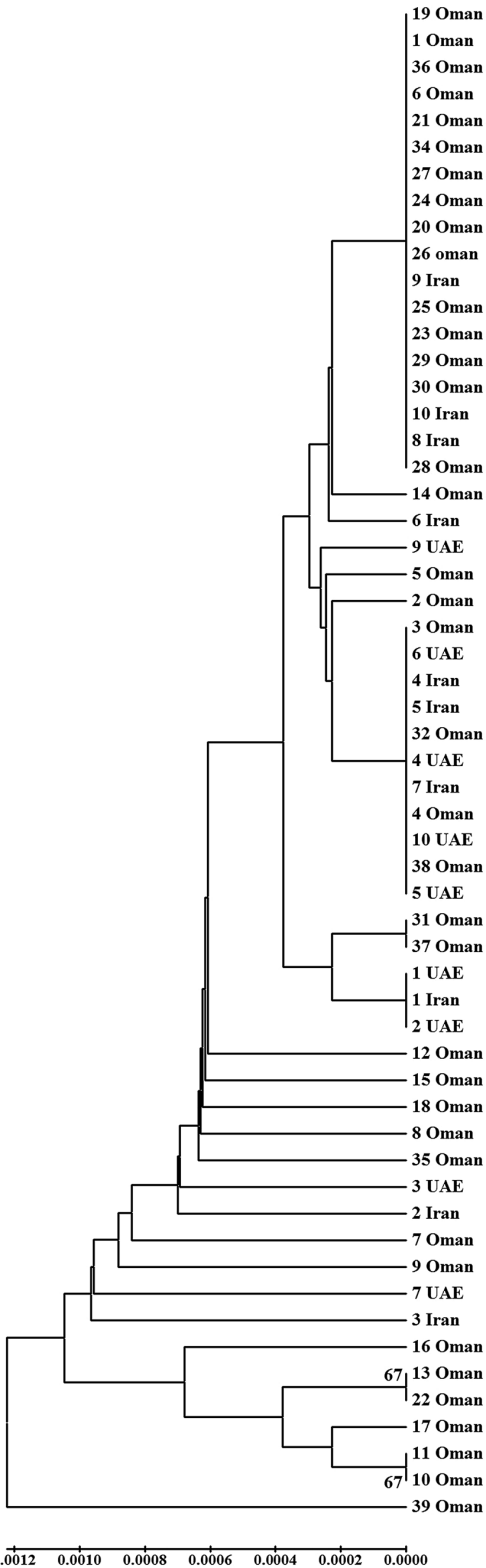
UPGMA tree showing the analysis of 57 phytoplasma strains from Oman, the UAE and Iran based on the concatenated sequences of 16S rRNA, *secA* and *imp* genes. The total length of the concatenated sequences is 2239. GenBank accession numbers for the three genes of the 57 strains are listed in Table 1. The tree was prepared based on the matrix of pairwise distances using the Kimura 2 parameter evolutionary model. Bootstrap values above 50 % are shown (1000 replications). All positions containing gaps and missing data were eliminated. The units indicate the number of base substitutions per site

Phylogenetic analysis of the 57 strains based on the concatenated sequence of the 16S rRNA, *sec*A and *imp* genes (2239 bp) showed clustering of most strains from Oman, the UAE and Iran. There was no relationship between clustering of the strains and the countries of origin or the regions in which they were collected (Fig. 5). The overall divergence among and within the Omani, Iranian and UAE sub-populations was found to be 0.001.

## Discussion

Witches’ broom disease symptoms were observed in all the surveyed regions in Oman, UAE and Iran. Analysis of diversity among 57 phytoplasmas from Oman, UAE and Iran based on sequences of the 16S RNA gene showed that all share a high level of nucleotide similarity (mean: 99.7 %).

Previous studies detected limited variation in the 16S rRNA gene sequences among phytoplasma strains belonging to the same group (Bertaccini and Duduk 2009). The findings of this survey indicated the existence of a limited variation among ‘*Ca.* P. aurantifolia’ strains from Oman, UAE and Iran, not only based on the 16S rRNA gene, but also on *secA* and *imp* genes. None of the sequence of the three genes separated strains based on the country from which they were obtained. This finding, together with the overall low level of genetic divergence, may indicate that phytoplasma strains from the three countries have the same origin and could have been moved among the three countries via infected propagation material (Al-Sadi et al. 2012b). It is therefore important to inspect propagative material of acid lime for phytoplasma infection using appropriate detection tools (Duduk et al. 2013; Al-Sadi et al. 2012b). Trade is very active among the three countries and the exchange of agricultural material and products is very common owing to globalized market (Al-Sadi et al. 2012a, 2013). It is possible that the phytoplasma moved from the northern part of Oman to the UAE due to trade activities and germplasm exchange between families living on either side of the countries’ borders. In addition, the active trade between the northern part of Oman and the southern part of Iran could have contributed to moving infected material between the two countries. Additionally, the insect vector of WBDL (*Hishimonas phycitis*) and its close relationship with lime (or with some other citrus plants) may have provided a unique and narrow ecological niche for the WBDL phytoplasma resulting in its low genetic divergence.

Analysis of divergence among 57 isolates from the three countries showed that *imp* is highly conserved compared to the 16S rRNA and *secA* genes. Siampour et al. (2012) reported variation based on the *imp* gene and the presence of three differentiable subgroups when 18 ‘*Ca.* P. aurantifolia’-related strains were analyzed (*imp*-A, *imp*-B and *imp*-C). In their study, the strains were obtained from a range of different host plant species such as alfalfa, tomato, eggplant, carrot, periwinkle, pear, peanut and others, together with only one samples from lime (Siampour et al. 2012). The very low variability of the *imp* gene found in this work is in agreement with *imp* gene reported feature as a phytoplasma gene subjected to positive environmental selection (Kakizawa et al. 2009). It also indirectly confirms the possible spreading mainly through propagation materials (Al-Sadi et al. 2012b). *SecA* gene has been reported to give high resolution among the different ribosomal groups (Bekele et al. 2011), however also in this gene sequences a very low level of divergence among phytoplasma strains was detected.

## Conclusion

The genetic diversity of ‘*Ca*. P. aurantifolia’ in Oman, UAE and Iran was analyzed for the first time based on three genes (16S rRNA, *imp* and *secA*). Data provided evidence of low genetic diversity among all strains based on analysis of the three genes. Future studies should target the association of other ‘*Ca*. P. aurantifolia’ genes with geographical locations and any symptoms that are not typical of WBDL.

## Methods

### Survey and collection of samples

**Fig. 6:**
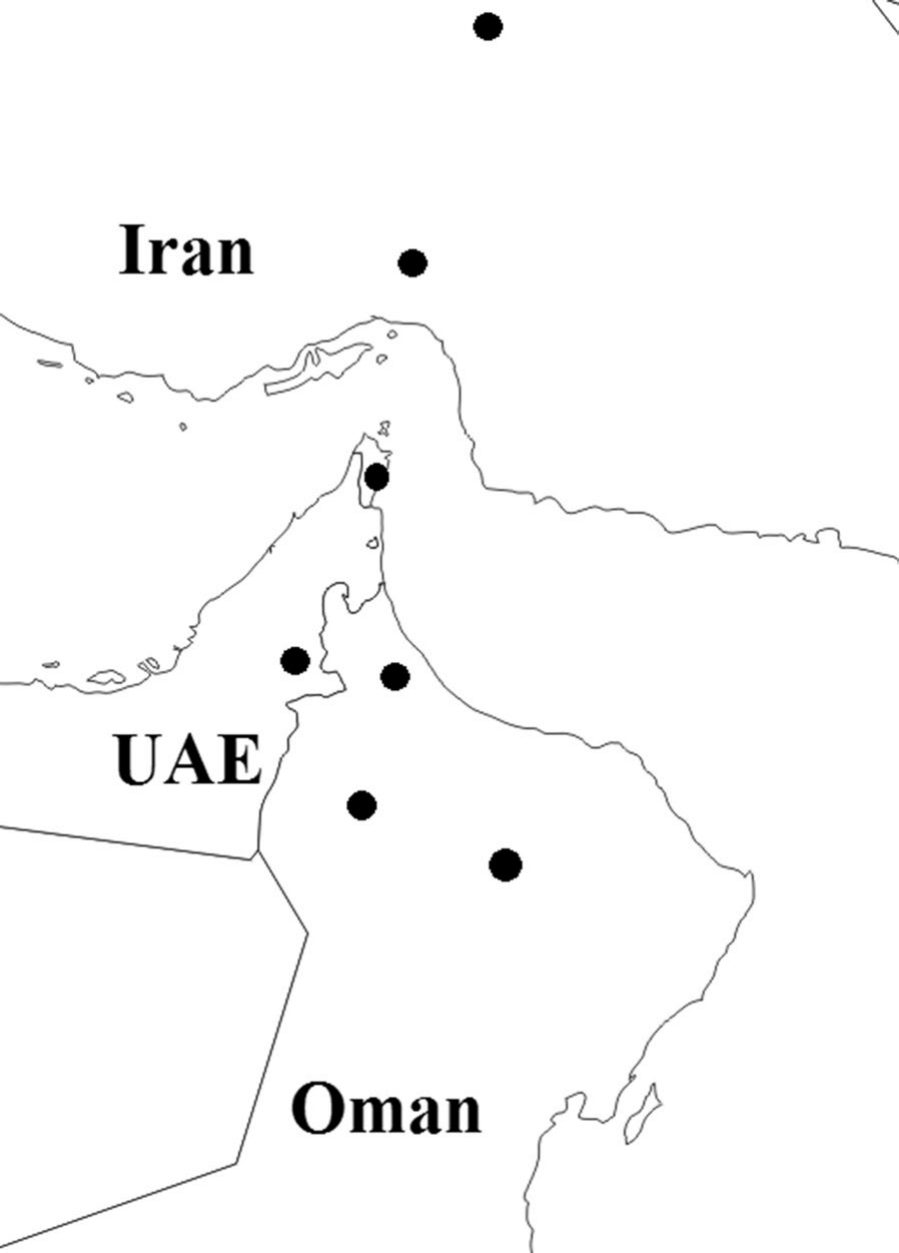
A map showing regions in Oman, the UAE and Iran from which samples were collected (indicated by *bold circles*)

Acid lime leaf samples were collected in Oman, UAE and Iran from trees with typical WBDL symptoms (Figs. 1, 6). Samples were collected over November 2013 to March 2014. Each sample consisted of at least 30 g of leaves which were kept in a plastic bag and stored in a cool box. All sampling details including location of the farm and age of trees were recorded.

In Oman, samples were collected from 4 governorates: Musandam, Batinah North, Dakhilya, and Dhahira (Fig. 6). At least 15 samples were collected from 5 farms in each governorate. Lime leaf samples were also collected from 6 farms in Al-Ain (UAE) in March 2013. In addition, samples were collected from Hormozgan and Kerman (Iran) (Fig. 6). At least 20 samples were collected from each country. All samples were labeled and transferred to Plant Pathology Research Laboratory, Sultan Qaboos University, where they were stored at −80 °C until used.

### DNA extraction

Lime leaves were washed with tap water and disinfected by 70 % ethanol to remove contaminants. About 1 g of leaf midribs was ground using liquid nitrogen in sterilized mortars and pestles and DNA extraction was carried out by using DNeasy Plant Mini Kit (QIAGEN, GmbH, Hilden, Germany) according to manufacturer’s instructions.

### Polymerase chain reaction (PCR)

Detection of phytoplasma in the samples was done using the universal primer pair P1 and P7 (Deng and Hiruki 1991; Schneider et al. 1995) (Table 1). PCR was done by using the following conditions: 94 °C for 30 s, then 35 cycles of 95 °C for 2 min, 53 °C for 60 s and 72 °C for 90 s, and final extension of 72 °C for 10 min (Sharmila et al. 2004). The reaction consisted of PuReTaq™ Ready-To-Go PCR™ beads (HVD Life Sciences, Vienna, Austria), 0.4 µM of each primer, ~25 ng DNA and sterilized distilled water up to 25 µl. Nested PCR using the primer pair R16R2 and R16F2n was carried out as described by Gundersen and Lee (1996). PCR products were separated on 1 % agarose gel in TBE buffer containing 1.5 µl of ethidium bromide and visualized under UV light.

The 57 DNA samples positive to phytoplasma were subjected to further PCR analysis to amplify s*ecA* gene by direct and semi-nested PCR assays using SecAfor1, SecAfor2, and SecArev3 as explained by Hodgetts et al. (2008). The conditions of PCR were 94 °C for 2 min followed by 35 cycles of 94 °C for 30 s, 53 °C for 60 s and 72 °C for 90 s and final extension of 72 °C for 15 min. First-round PCR product was diluted 1: 200 with sterilized distilled water and 1 µl of diluted product was used in semi-nested PCR using the primer pair SecAFor2/SecArev3 and the same reaction mixture and PCR conditions described above (Table 1). PCR products were separated as described above.

Amplification of *imp* gene was done using primers ImpF and ImpR as described by Siampour et al. (2012) (Table 1). The amplification and detection were carried out as described previously.

### Sequence analysis

PCR products of the 16S rRNA, *secA,* and *imp* genes for the 57 samples collected from Oman, UAE and Iran were directly sequenced at Macrogen, Korea using the same primers employed for their amplification (Table 1). Sequences were aligned using Clustal-W program in Chromas Pro (version 1.41; Technelysium Pty Ltd, Brisbane, QLD, Australia). Construction of UPGMA tree was done based on the matrix of pairwise distances using the Kimura 2 parameter evolutionary model (Mega 6) (Tamura et al. 2013). Sequences of the 16S rRNA, *secA* and *imp* genes of reference strains of ‘*Ca*. Phytoplasma aurantifolia’ and phytoplasmas from other 16Sr groups obtained from National Center for Biotechnology Information (NCBI) were used for comparison with sequence from this study. Bootstrap consensus trees were generated based on 50 % majority-rule using 1000 replications. In addition, genetic divergence was calculated based on the number of base differences per sequence from averaging over all sequence pairs between groups and all ambiguous positions were removed for each sequence pair (Tamura et al. 2013).
